# Comprehensive management for complex complications after periumbilical fat implantation into the upper eyelid

**DOI:** 10.1186/s12886-023-02989-z

**Published:** 2023-07-10

**Authors:** Xuewei Jiang, Wei Chen, Nan Chen, Yu Wang, Jiayan Lin, Xufeng Tian, Hailong Wu, Qun Zhang

**Affiliations:** 1 Department of Plastic and Reconstructive Surgery, Shanghai Ninth People’s Hospital, Shanghai Jiao Tong University School of Medicine, Shanghai, China; 2 Department of Plastic Surgery, Mylike Medical Cosmetic Hospital, Shanghai, China; 3 The Affiliated Eye Hospital, Nanjing Medical University, Nanjing, Jiangsu China; 4 Department of Plastic and Reconstructive Surgery, The First Affiliated Hospital of Anhui Medical University, Hefei, Anhui China; 5 Department of Plastic and Reconstructive Surgery, The Frist Affiliated Hospital of Harbin Medical University, Harbin, Heilongjiang China; 6 Department of Plastic and Reconstructive Surgery, Shanghai Tenth People’s Hospital, Tongji University, Shanghai, 200072 China; 7 Department of Plastic Surgery, NuoYa Stomatological Hospital, Siping, Jilin China

**Keywords:** Periumbilical fat grafting, Upper eyelid, Blepharoplasty, Postoperative deformity, Pressure sensation

## Abstract

**Background:**

Recently, periumbilical fat (PF) mass, an autologous material with a high survival rate, has been transplanted to treat sunken or dissatisfactory double eyelids. However, the intricate complications of PF grafts and associated reconstructive strategies are infrequently discussed.

**Methods:**

During 3 years, 20 patients (33 eyes) with eyelid malformations caused by PF grafts into the orbital septum or on the surface of the levator aponeurosis underwent corrective blepharoplasty. We recorded patients’ subjective feelings and identified deformities from crease abnormalities, bloated appearance, and problems with the eyelid’s height. Then, we categorize them into three types based on their complexity: type I, swollen appearance; type II, obvious adhesion; type III, severe comprehensive damage. The relevant management included removing fat implants, releasing the adhesion, and rebuilding the physical structure according to the anatomic damage mechanism. The improvement effect was assessed with a satisfaction survey from patients and doctors at 6 months of follow-up.

**Results:**

The swollen appearance was observed in 26 eyes (78.8%), an unsmooth double-eyelid line was in 23 eyes (69.7%), and the incidence of adhesion was in 22 eyes (66.7%). Following a comprehensive evaluation, 15 eyes (45.5%) and 13 (39.4%) were classified as type I and type II respectively. After the 6-month follow-up, 22 eyes (66.7%) showed exceptional aesthetic results, whereas only 2 eyes as type III had a poor outcome.

**Conclusions:**

The deformities emerging from periumbilical fat into the upper eyelid are associated with the shape of the fat and the adhesion in tissues. Graft removal, adhesion release, and restoration of the natural anatomic structure can have positive outcomes.

## Introduction

Eyelids, the most prominent area of the human face, can expose early signs of aging. The primary clinical manifestations of eyelid aging include hollowness, skin laxity, and crease, composing the fatigued appearance [1–3]. Loss of fat volume caused by atrophy or blepharoplasty is considered the potential contributing factor to sunken deformity in upper eyelids [4, 5]. Therefore, filling the deficient anatomic structure with autologous fat, an easily accessible autogenic substance, has grown in popularity over traditional subtractive surgical operations for eyelid rejuvenation [6].

In the common autologous fat grafting technique, the fat granule harvested from the abdomen or other donor sites was injected into the hollow eyelids by small cannulas [7–9]. Postoperative results are variable, depending on the survival rates of fat grafts. Other complications such as ptosis, lipogranuloma, and severe vascular embolism have also been reported [10–13]. Aiming to reduce the risk and raise postoperative satisfaction, periumbilical fat (PF) mass was transplanted as a new fat supplementation into the compartment between the orbicularis oculi muscle and the levator palpebrae superioris (LPS) in recent research [14–16]. However, eyelid deformities after PF grafting have been rarely reported. Over the past 3 years, we repaired 20 patients’ eyelids (33 eyelids) with PF blocks in the upper eyelid for more than six months. The purpose of this study was to observe the features of these complex deformities and evaluate the specific management based on anatomical structure injury.

## Materials and methods

A total of 20 patients (33 eyes) with deformities related to periumbilical fat transplantation underwent the repair operation and continuous follow-up for 6 months in Shanghai Mylike Medical Cosmetic Hospital between October 2018 and September 2021. We collected patients’ medical histories, preoperative examinations, and postoperative surveys. Inclusion criteria included: (1) the interval between PF transplant and this repair was more than 6 months and less than 1 year; (2) being older than 18 years of age; (3) voluntary participation in treatment and compliance with follow-up. Patients with systemic diseases, congenital levator dysfunction or myasthenic ptosis, secondary upper blepharoplasty or revision surgery, severe psychological problems or mental disorders, and refusing to participate in or disagreeing with treatment plans were excluded. The same photographer photographed all patients in our studio during the study. The photographs were taken in a fixed position 1.5 m from the patients. The same professional surgeon performed corrective blepharoplasties; other surgeons completed previous transplanted operations on all patients. Finally, we carried out a satisfaction survey (5-point Likert scale) judged by each patient herself and a doctor blinded to the study at the 6-month follow-up. Then, we defined excellent as satisfactory effects (rating ≥ 3) from both doctor and patient, fair as satisfaction (rating ≥ 3) only from one, and poor as unsatisfactory effects (rating ≤ 2) from them. The satisfaction rate was calculated as the proportion of eyes scoring an average ≥ 3.5. The study was approved by the ethics committee of our institution and conducted under the principles of the Helsinki Declaration. All patients gave informed consent before study entry.

### Preoperative evaluation and classification

Preoperative evaluation of the periorbital region necessitated experienced surgeons and was critical in designing the repair operation scheme. It was a comprehensive result of the present deformity and primary personal condition. Depending on the degree of eyelid anatomical structure and function damage caused by the graft, the deformity manifested in a variety of complex patterns. While there was no report about it, we identified the malformation based on the common complications related to double eyelid surgery [17]. In our observation, crease abnormalities and swollen appearances were the main esthetic problems, as well as the patient’s dissatisfaction. So, the focus of the evaluation system included an unsmooth or descending curve of the double-eyelid crease, eyelid swelling, multiple folds, blepharochalasis, and the extent of adhesion.

None of the patients was diagnosed with obvious ptosis, and the function of LPS was difficult to assess due to interference from transplants and tissue adhesion. However, the complaint, which was described as a sense of pressure with the eyes open, was one of the main reasons for seeking medical care. In our view, it could not be ignored because it might indicate injury to LPS function from subjective sensation.

To make precise and overall assessments, we tried to obtain the patient’s preoperative photographs and compare them with current conditions, if possible. Meanwhile, we categorized the complicated complications into three types: type I - swollen appearance, type II - obvious adhesion, and type III - severe comprehensive damage (Table 1).

**Table 1:** Classification of complications and strategy of the repair

Type	Eyes, n (%)	Features			Intraoperative condition	Strategy of the repair
		Swelling	Adhesion	Subjective sense		
I	15 (45.5%)	Obvious	None/Slight, at medial	With/Without heaviness	Excessive grafts	Remove the transplants
II	13 (39.4%)	Slight	Obvious	With/Without tension	Structural adhesion	Release the adhesion
III	5 (15.2%)	Obvious/ Blepharochalasis	Obvious/ multiple folds	With/Without complex or serious sense	Anatomical disorder	Restore the structure

I, swollen appearance; II, obvious adhesion; III, severe comprehensive damage

***Type I, swollen appearance***, obvious bulky upper eyelid, especially during eye closing; slightly unsmooth double-eyelid line; the adhesion is slight and located at the medial eyelid; little scar without obvious adhesion above the crease; heavy sensation as the main expression in the sense of pressure.

***Type II, obvious adhesion***, obvious adhesion above the crease or other positions not confined at the medial, which might have a great effect on the appearance of the eyelid, including double-eyelid crease; obvious scar above crease caused by ectopic adhesion between the skin and the LPS aponeurosis; slightly swollen appearance; tensional sensation as the main expression in the sense of pressure.

***Type III, severe comprehensive damage***, obviously swollen subcutaneous soft tissue and adhesion which might affect the appearance of the eyelid deeply; other features (multiple folds, blepharochalasis); more complex or more severe discomfort.

Considering the patients’ expectations and anatomic complicated changes, we combined types of complications with intraoperative conditions to design the corresponding target of the repair, respectively: removing the transplants, releasing the adhesion, and restoring the structure beyond removing the transplants and adhesion (Table 1). Finally, some surgical procedures were adjusted according to the actual structural damage.

### Surgical technique

#### Incision Design

Before the operation, the surgeon marked the obvious swelling of the eyelid when the patient was standing and repeatedly measured the amount of loose skin by stretching the upper eyelid skin when opening and closing their eyes. It was critical to remove the skin conservatively according to the degree of loosening because the volume of the eyelid, the main factor in loose skin, would change after the repair. The inferior incision was designed along the scar as possible to reduce skin damage.

### Repair procedures

A local anesthetic (mixture of 0.5% lidocaine and 1:200,000 epinephrine) was injected into the skin slowly and infiltrated into the adhesion. Then the skin was incised along the predesigned curves; the scar adhesion should be released carefully to avoid secondary damage, particularly to the LPS aponeurosis and tissue anterior to the tarsal plate.

**Fig. 1:**
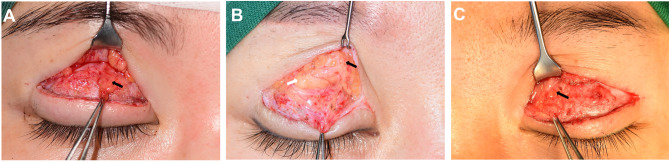
Intraoperative figures of patients. **a** The figure of a type I patient. Periumbilical fat mass with a high survival rate was on the surface of the levator palpebrae superioris (black arrow). The adhesion between tissues and fat mass was slight. **b** The figure of a type II patient. The fibrous tissue is tightly surrounded periumbilical fat (black arrow), retro-orbicularis oculi fat, and levator palpebrae superioris. The boundary between grafts and septum fat (white arrow) was vague. **c** The figure of a type III patient. Identifying grafts and tissues was more difficult caused of heavy adhesion formation. Periumbilical fat was hidden in the disordered mixture (black arrow)

In the eyelid of the type I complication, the transplanted PF with high survival adhered to the septum fat and the LPS aponeurosis marginally (Fig. 1a). The function of the LPS was well detected. After releasing the slight adhesion, plenty of PF was removed, and residual fat mass was laid flat on the surface of the aponeurosis. After completing these steps, some patients expressed that the feeling of pressure disappeared when opening their eyes. In some severe sunken or septum fat deficiency cases, we adjusted the trimmed PF at the medial septum to fill the volume or reconstruct the glide zone. The border of the PF was sutured between the residual orbital septum and the upper margin of the tarsus with 6 − 0 nylon.

In the eyelid of the type II complication, the PF adhered tightly to the septum fat, retro-orbicularis oculi fat (ROOF), and the LPS aponeurosis (Fig. 1b). It was an essential step in clearly identifying the tissues and grafts from the complicated mixture before completely loosening the adhesion. Before untangling the anatomical structures, we would not easily resect the seemingly redundant tissues, especially around the LPS. Injecting a little saline into the interstitial space can make the fibrous tissue sparse and distinct from the functional structures. Like type I, the sense of pressure disappeared after blunt dissection. Then we would trim the extra transplanted fat, which might be restricted by the adhesion, to improve the swollen appearance of the eyelid, if necessary. Further, to prevent the recurrence of adhesion, we released and transferred lateral septum fat to cover the adhesion site above the grafts. When the septum was lacking in fat, homologous fat from the other upper eyelid or the lower eyelid was the ideal material to block adhesion formation.

In the eyelid of the type III complication, the PF adhered extensively to the septum fat, the ROOF, the LPS aponeurosis, and the orbicularis oculi muscle (OOM) (Fig. 1c). The damage to anatomical structure and function was worse than for the other two types and increased the difficulty of identifying the tissue, even if the fat mass might be less. Releasing the adhesion and removing PF sufficiently might help to avoid the formation of new adhesion and secondary injury to the tissue, especially the LPS. In this situation, we also paid more attention to maintaining the integrity of the ROOF. We distributed ROOF into the gliding system as double protection with septum fat, which was better than the other grafts. Furthermore, we also considered that restoring the normal anatomical position of the tissues can effectively reduce deformities after operations.

### Postoperative care

Ice was applied on the surgical eyelid intermittently within 24 h after surgery. Oral antibiotics were used for three days as normal, and the surgical sutures were removed after 7 days.

## Results

Twenty patients with an average age of 33.1 years (ranging from 27 to 47 years) were involved in our study, all of whom were Asian females. In total, 33 eyelids were repaired and followed up on. The preoperative evaluation showed that a swollen appearance with 26 eyes (78.8%) was the most common deformity among these complications, and 23 eyes (69.7%) suffered from an unsmooth double-eyelid line which was close to the incidence of adhesion (22, 66.7%). Severe symptoms, including multiple folds (3, 9.1%) and blepharochalasis (2, 6.1%), were significantly less than the other three. From the aspect of the subjective sense, 18 eyes (54.5%) presented a sense of pressure when open and closed. Other complications, such as eyelid masses and ptosis, were not observed in these cases.

**Fig. 2:**
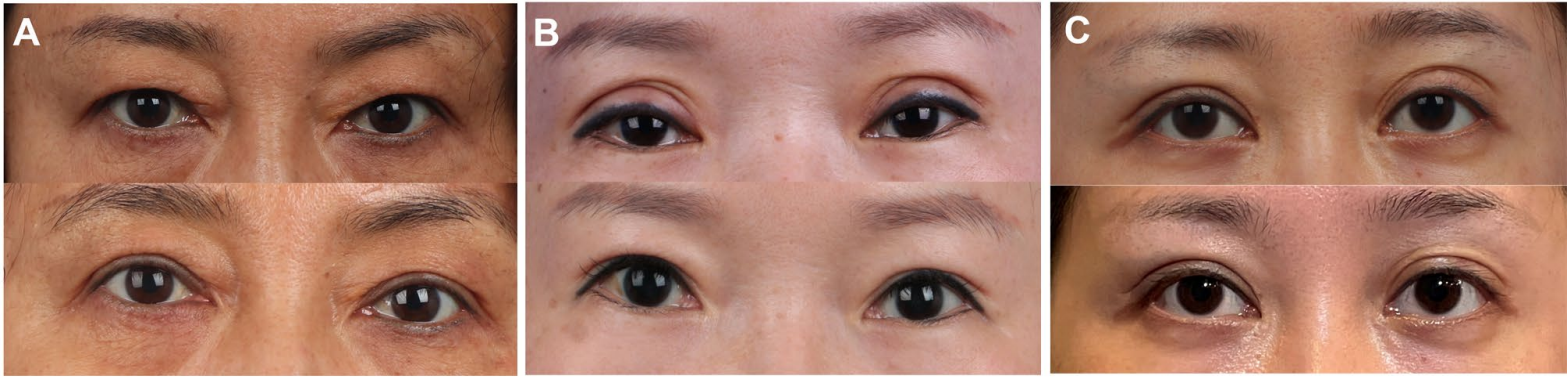
Preoperative and postoperative photographs of patients. **a** A 47-year-old woman with type I complications received corrective blepharoplasty for bilateral eyelids. The preoperative view showed an obvious swollen eyelid appearance, even covering the double-eyelid line. **b** A 39-year-old woman with type II complications underwent a repair operation for both eyes. The preoperative photograph showed obvious adhesion above the crease and beyond the medial of the eyelid. **c** A 32-year-old woman with type III complications underwent repair surgery for the left eyelid after periumbilical fat transplantation into bilateral eyelids. The preoperative view showed obvious multiple folds on the left eyelid

At the 6-month follow-up, the satisfaction rate was 51.5%. After classification, 22 eyes (66.7%) received excellent results, 9 eyes (27.3%) were fair, and 2 eyes (6.1%) received poor outcomes. The improvement of the deformities in type I gained high approval, with 13 eyes (86.7%), while 40% of type III eyes had a poor outcome (Table 2; Fig. 2).

**Table 2:** Satisfaction survey

Type	Eyes, n	Excellent (%)	Fair (%)	Poor (%)
I	15	13 (86.7)	2 (13.3)	0 (0)
II	13	7 (53.4)	6 (46.2)	0 (0)
III	5	2 (40)	1 (20)	2 (40)
Total	33	22 (66.7)	9 (27.3)	2 (6.1)

I, swollen appearance; II, obvious adhesion; III, severe comprehensive damage

**Fig. 3:**
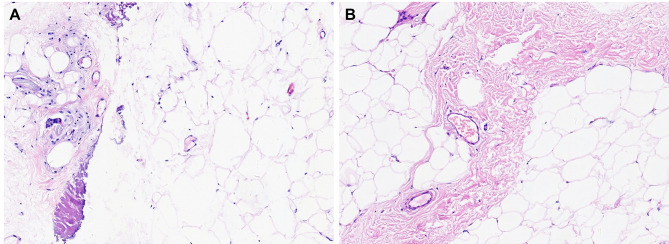
Histopathology of patients showed normal adipocytes and mild local fibrosis without obvious adiponecrosis and neutrophil infiltration (hematoxylin and eosin × 100). **a** The section from a type I patient. **b** The section from a type III patient

After obtaining the consent of two patients with malformation types I and III, the biopsy showed fibrous proliferation with infiltration of lymphocytes and plasma cells. In contrast, fat necrosis, and neutrophil infiltration were not found (Fig. 3).

## Discussion

More Asians with single eyelids receive blepharoplasty to build charming double eyelids to satisfy increased aesthetic demands for appearance. In contrast to Caucasian upper eyelids which have a high fold and depressed shape, Asian eyelids are bloated and droopy due to anatomic structure, such as hypertrophy septum fat and insufficient terminal LPS aponeurotic fibers interdigitated with pretarsal skin and OOM [18, 19]. Therefore, excising the prolapsed septum fat is key to forming a stable and deep fold in traditional Asian blepharoplasty [20]. However, iatrogenic eyelid depression is a major reason for surgery failure due to inadvertent excessive removal of excessive fat. In addition, the atrophy of fat associated with aging also contributes to sunken eyelids [17, 21]. To improve the depressed appearance, diverse biological materials are applied to fill in the upper eyelids, such as orbital fat flap, free fat graft, dermis-fat graft, hyaluronic acid fillers, etc. [22–24]. However, fat flap transposition and hyaluronic acid injection might not be enough to correct the severe sulcus deformity (Grade 3 of the Park S classification [25]: a sunken depth of 1 cm or more). Although fat granules injection was accepted widely due to its advantages, like easy access, less trauma, and no potential hypersensitivity [13]. But it was highly technique dependent and uncertain absorption rate, ranged from 20 to 90% [26]. Besides, blind injection via canula or needle could easily cause underlying structural damage and even blindness or stroke [13]. Dermal fat with a higher survival rate is prone to blepharoptosis [27].

Currently, periumbilical fat, the normal material in eye socket reconstruction, is exploited in blepharoplasty. Compared with other frequent harvesting sites like the buttock and the flank, the periumbilical area is more concealed, which can avoid visible scars on the body [28]. PF is composed of fat mass and small vascular vessels. Thus, in severely sunken cases, some surgeons transplanted PF into the ROOF layer or the orbital septum to augment the contents volume and achieved satisfactory results [14–16]. Wang et al. considered that it might replace the missing septum fat to reconstruct the sliding system between LPS and OOM in repairing failed double-eyelids [16]. However, 20 patients (33 eyelids) who received PF grafts into their upper eyelids came to our department and required repair surgery. We classified the morphological characteristics of their eyelids based on common long-term complications after blepharoplasty and analyzed the relevant anatomic structural damage.

The dissatisfactory post-transplant presentations were primarily attributed to a swollen appearance (78.8%) and/or tissue adhesion (66.7%). In type I cases with obvious bulky eyelids, we observed that the mass was especially prominent when closed and drooped to cover the double-eyelid line slightly when opened. Some patients complained pressure when they tried to widen their eyes, and even some felt uncomfortable when opening their eyes normally. Though the diagnostic tests for blepharoptosis were not positive, we still believed that the weighty fat mass impacted the function of LPS, and the noticeable signs would emerge from the long-term LPS damage. Surgery operations also verified this hypothesis that the perceived stress disappeared after removing plenty of grafts. From this aspect, controlling the shape and volume of PF accurately might be a critical factor for the transplantation, especially for the load of LPS. Additionally, we preferred the lamellar PF over the thick mass to avoid the overcorrection of the sunken superior sulcus. It was easy to exaggerate the loss of fat volume if the surgeon ignored the dynamic changes in upper eyelid morphology accompanying eye movements. Moreover, we observed that PF survived well in the orbital septum, similar to the conditions in the ROOF layer that Zhou reported [14]. It also suggested that the volume of PF should be conservatively estimated before transplantation, different from excessively injecting fat granules with a high absorption rate.

Adhesion formed by fibrous connections between PF grafts and the surrounding tissue was the other potential mechanism for the complex deformities. The extent of adhesion also affected the anatomic structure’s physiological function, even promoting the absence of a glide zone in serious situations. In type II and some type III cases, OOM, septum fat, and LPS were restricted by graft adhesion. It led to an imbalanced distribution between the force point of LPS and lower skin flap inferior of the incision, which contributed to obvious creases and the double-eyelid lines’ unsmooth and indented scars. Combined with the pressure complaint, we used the spring model to explain the mechanical injury of LPS caused by adhesion. The adhesion strengthened the pulling force to LPS from skin and PF, just as Kim’s spring model theory revealed the relationship between the increasing weight load on LPS and a high fold [29]. Therefore, we stressed that releasing the adhesion completely and restoring the tissues’ physiological position was beneficial for preventing ptosis and improving the deformities. Further, in our experience, reconstructing the gliding system was an effective solution to reduce the recurrence of adhesions. After removing the grafts, we transferred the residual septum fat flap to cover the adhesion area. We folded the local fat flap to repair the broken ROOF if necessary. Because we believed that the integral ROOF and septum fat, normal tissues in the eyelid, were essential ingredients for the glide zone to avert adhesion between the skin and LPS, rather than depending on various grafts. The general satisfaction rate after operations also supported this view.

Interestingly, the histopathology of PF grafts showed slight inflammatory cell infiltration and localized fibrosis, contrary to the feature of lipogranuloma after fat injection. In other words, PF might produce a milder inflammatory response than fat granules or other fillers. Meanwhile, obvious fibrous proliferation around fat cells might reveal the pathological mechanism of adhesion formation after transplanting PF.

The limitations of this study should be noted. The number of cases is small, and the results are primarily subjective. To comprehensively evaluate the effect of this technology, we will collect and analyze the data of patients with PF into ROOF if possible. Even more to the point, based on our preoperative judgements on anatomic injury and worse correlation between grafts and natural tissues, we preferred to choose surgical treatments than using cannulas blindly. It could help us understand the mechanism of deformities visibly and got satisfied outcomes which complied to patients’ willingness. So, our results were only for invasively managing complications of PF transplantation. The more comparative studies are essential to determine different treatments and filling techniques, such as invasive surgery and injection by cannulas.

## Conclusion

Periumbilical fat grafted into the upper eyelid may cause complex aesthetic problems, including a swollen appearance, an unsmooth double eyelid line, and adhesion. The fundamental reasons for these deformities are adhesion and structural damage caused by fat mass. Removing grafts and releasing the adhesion completely favor reconstructing the physiological functions of normal tissues and producing satisfactory aesthetic effects. It suggests that the technique for grafting periumbilical fat into the upper eyelid should be refined. Controlling grafts’ size and shape precisely and selecting suitable implant locations might avoid secondary injury.

## Data Availability

The datasets generated and/or analyzed during the current study are not publicly available due to privacy and ethics but are available from the corresponding author on reasonable request.
